# Rehabilitation following shoulder arthroplasty: a survey of current clinical practice patterns of Italian physiotherapists

**DOI:** 10.1186/s40945-023-00166-5

**Published:** 2023-06-05

**Authors:** Fabrizio Brindisino, Mariangela Lorusso, Michele Usai, Leonardo Pellicciari, Sharon Marruganti, Mattia Salomon

**Affiliations:** 1 Department of Medicine and Health Science “Vincenzo Tiberio”, University of Molise, Campobasso, Italy; 2 Department of Clinical Science and Traslational Medicine, University of Roma “Tor Vergata”, Rome, Italy; 3 “Sardo” Private Practice, Cagliari, CA Italy; 4 IRCCS Istituto delle Scienze Neurologiche di Bologna, Bologna, Italy

**Keywords:** Shoulder prosthesis, Physical therapy modalities, Total shoulder replacement, Shoulder joints, Physical therapy specialty

## Abstract

**Background:**

The incidence of Total Shoulder Arthroplasty (TSA) and Reverse Total Shoulder Arthroplasty (RTSA) is constantly increasing. As a result, the interest in post-surgical rehabilitation has grown, since it is crucial in order to achieve full recovery and successful outcomes. The first aim of this study is to investigate the Italian physiotherapists (PTs) clinical practice in the management of patients with TSA and RTSA and to compare it with the best evidence available in the literature. The second purpose of this study is to assess any existing difference between the survey answers and the different sample subgroups.

**Materials and methods:**

This cross-sectional observation study was designed following the CHERRIES checklist and the STROBE guidelines. A 4-sections survey with a total of 30 questions was developed for investigating post-surgery rehabilitation management in patient with TSA and RTSA. The survey was sent to Italian PTs from December 2020 until February 2021.

**Results:**

Six-hundred and seven PTs completed the survey regarding both TSA and RTSA; 43.5% of participants (*n* = 264/607) stated that TSA is more likely to dislocate during abduction and external rotation. Regarding reverse prosthesis, 53.5% (*n* = 325/607) affirmed RTSA is more likely to dislocate during internal rotation, adduction and extension.

In order to recover passive Range of Motion (pROM), 62.1% (*n* = 377/607) of participants reported that they gain anterior flexion, abduction, internal rotation, external rotation up to 30°, with full pROM in all directions granted at 6–12 weeks. Regarding the active ROM (aROM), 44.2% (*n* = 268/607) of participants stated that they use active-assisted procedures within a range under 90° of elevation and abduction at 3–4 weeks and higher than 90° at 6–12 weeks, with full recovery at a 3-month mark. Sixty-five point seven percent of the sample (*n* = 399/607) declared that, during the rehabilitation of patients with TSA, they tend to focus on strengthening the scapular and rotator cuff muscles, deltoid, biceps and triceps. Conversely, 68.0% (*n* = 413/607) of participants stated that, for the rehabilitation of patients with RTSA, they preferably focus on strengthening the periscapular and deltoid muscles. Finally, 33.1% (*n* = 201/607) of participants indicated the instability of the glenoid prosthetic component as the most frequent complication in patients with TSA, while 42.5% (*n* = 258/607) of PTs identified scapular neck erosion as the most frequent post-RTSA surgery complication.

**Conclusions:**

The clinical practice of Italian PTs effectively reflects the indications of the literature as far as the strengthening of the main muscle groups and the prevention of movements, which may result in a dislocation, are concerned. Some differences emerged in the clinical practice of Italian PTs, regarding the restoration of active and passive movement, the starting and progression of muscle strengthening and the return to sport (RTS). These differences are actually quite representative of the current knowledge in post-surgical rehabilitation for shoulder prosthesis in the rehabilitation field.

**Level of evidence:**

V

**Supplementary Information:**

The online version contains supplementary material available at 10.1186/s40945-023-00166-5.


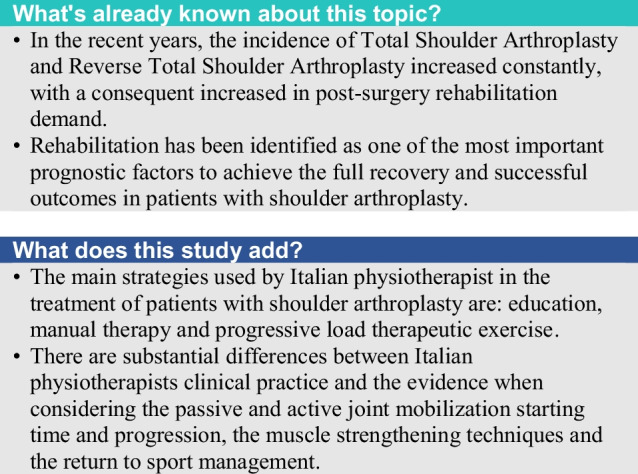


## Introduction

In the recent years, the incidence of Total Shoulder Arthroplasty (TSA) and Reverse Total Shoulder Arthroplasty (RTSA) is constantly growing, with a subsequently increased post-surgery rehabilitation demand [1–3].

To date, in orthopaedics the prevalence of shoulder arthroplasty ranks second, after hip and knee replacements [4, 5]. In Italy, shoulder replacement represents 4.5% of all arthroplasty types [6].

The main goal of shoulder arthroplasty is to reduce the patient’s pain and to improve the joint range of motion (ROM), by restoring or modifying the shoulder biomechanics and kinematics [7, 8]. The TSA surgery is widely recognized as the treatment of choice for patients with glenohumeral joint diseases, such as osteoarthritis, rheumatoid arthritis, osteonecrosis and proximal humerus fractures, when conservative treatment is not indicated [9–12]. Either way, in case of patients with significant Rotator Cuff (RC) involvement and anatomical bone defects, the first indicated treatment remains RTSA. RTSA is also suggested for the patients with eccentric osteoarthritis, displaced fractures of the proximal humerus, TSA revision surgery, rheumatoid arthritis and neoplasms of the humeral head [9, 13, 14]. In the recent years, the progressive and extensive use of TSA and RTSA is linked to its low risk of developing complications (i.e., 10.3 and 16.1%, respectively) [15, 16] and to the concurrent biotechnology innovations [17].

However, this trend does not seem to match the development of post-surgical rehabilitation management, which has been identified as one of the most important prognostic factors contributing to achieve full recovery and successful outcomes [18, 19] as the quantity of high quality evidence regarding the best choice of rehabilitation strategies after shoulder arthroplasty is limited [7].

There are significant differences between current post-surgical rehabilitation programs, which are essentially based on experts’ opinion [5, 8, 9, 12, 20, 21]. Notably, only few randomized controlled clinical trials concerning physiotherapy best practice are available [22].

In this context, the main purpose of this study is to analyse the clinical practice of Italian physiotherapists (PTs) in the rehabilitation of patients after TSA and RTSA surgery and to compare it with the best evidence available in the literature. The second aim of this study is to assess any differences between survey responses and different subgroup of the sample (i.e., clinical experience in years, academic background, degree in Orthopaedic Manipulative Physical Therapy (OMPT) and the number of patients with shoulder arthroplasty treated in one year).

## Materials and methods

### Study design

This cross-sectional observation study was designed following the CHERRIES checklist [23] and the STROBE guidelines [24]. The ethical approval was obtained from the Ethics Committee of Trento (Italy) with the registration number 6846-3. All the study-related procedures were performed according to the principles of the Declaration of Helsinki [25].

### Sampling and recruiting

This study involved only PTs working in Italy at the time of the survey, who spontaneously filled out the survey; as many participants as possible were recruited in a given period of time, according to other similar recent publications [26–29] and other international surveys [28–33].

All potential participants were invited to participate through a link, generated by the Survey Monkey® software and shared through various communication channels: a) social networks (Facebook and Twitter); b) instant messaging via mobile applications (WhatsApp and Telegram); c) e-mails, newsletter, and information channels of two professional associations, in particular the Italian Association of Physiotherapy -AIFI- and the Group of Manual Therapy and Musculoskeletal Physiotherapy -GTM-. In order to further increase the response rate, 6 reminders were sent through each means of communication (4 on weekly basis and the other 2 every 2 weeks). An electronic invitation form and an information letter were provided to all participants (Additional file 1 - Information letter).

### The best choice in TSA/RTSA rehabilitation management

The current best practice is inspired by the general rehabilitation principles, which are similar regardless of the type of shoulder prosthesis used. Overall, the evidence suggests promoting soft tissue healing and pain management, gently restoring the shoulder girdle muscle strength, its mobility and its endurance and optimizing functional recovery by promoting the patient’s responsibility during the treatment [8]. Therefore, the combination of therapeutic exercise, manual therapy techniques and a possible integration with patient’ education and counselling is strongly recommended [22].

In particular, for TSA, the rehabilitation progression is based on the acquisition of ROM and strength, taking into account the intrinsic variability of the length of the rehabilitation paths [12]. The use of shoulder sling for defined period of time is further recommended [34] being together with the protection of the subscapularis repair, of primary importance. Then, the gradual passive and active mobilization with introduction of active ROM management strategies varies from 4 to 7 weeks [8, 12, 35] with the resumption of normal activities between the third and fourth post-operative month [36].

In case of RTSA, a careful protection of the implant in early stages should be ensured, by avoiding combined movements of extension, adduction and humeral internal rotation, in order to prevent dislocations and to promote wound healing [8]; however, an early start of the rehabilitation program could represent a useful strategy to ensure a fast improvement [37]. The optimal choice in the management of the RTSA rehabilitation time is to continue the program for at least 4–6 months, with the aim of reaching 120° of active elevation and 30° of functional rotation [9, 38].

### Survey structure

The preliminary version of the questionnaire was structured after a careful review of the literature on rehabilitation management of patients with TSA and RTSA, and on TSA and RTSA postoperative complications. This literature review was conducted by two independent authors (ML and MS) with 5 years of experience in the rehabilitation of shoulder musculoskeletal disorders. In order to increase this questionnaire content validity, the preliminary version was evaluated by a team of experts, namely one of the authors of this survey (FB, with 15 years of experience in shoulder rehabilitation), a physician specialized in physical medicine and rehabilitation with 10 years of experience in shoulder disease management, an orthopaedic surgeon with 7 years of experience in shoulder surgery, and two patients who underwent TSA and RTSA surgery.

A panel of experts evaluated independently this questionnaire regarding the use of terms and the wording, the order of the question and the structure of the survey, in order to improve the survey as much as possible. In any case, this preliminary version did not undergo any substantial change after the experts’ assessment, since it met their general consensus.

In order to refine the questionnaire acceptability and comprehension of the, this survey was submitted to 20 PTs (ten with OMPT specialization), working in Italy. This poll of PTs was recruited to investigate the survey potential ambiguity, its clarity and the fairness of its questions. No ambiguity, formal flaws in writing nor difficulties in understanding the questions were detected.

Therefore, the authors confirmed the final version of this survey and they divided it into 4 sections, for a total of 30 multiple-choice questions.

The first section consisted of 7 questions (**Q1-Q7**) aimed at collecting demographic information (e.g., gender, age, setting and years of clinical experience), level of academic qualification and number of patients treated through shoulder arthroplasty assessed within 1 year of their clinical practice.

The second section consisted of 5 questions (**Q8-Q12**) regarding general information about post-surgery rehabilitation management of patients with TSA and RTSA (e.g., patient education, pain management, swelling management, early post-operative management).

The third and fourth sections both consisted of 9 questions, investigating in detail the management of the patients during the different rehabilitation stages and the possible TSA rehabilitation complications (**Q13-Q21**) and RTSA (**Q22-Q30**). The whole questionnaire was detailed in Additional file 2.

### Data collection

This survey was available on the Survey Monkey® platform (Survey-Monkey, Palo Alto, California, www.surveymonkey.com) for 2 months, from 21 December 2020 to 21 February 2021. At the end of the defined period, no further requests to complete this questionnaire has been registered by the authors.

This questionnaire could be only completed once per mail address, the participation occurred on a voluntary basis and no incentives were provided for its participants. Moreover, a mandatory written informed consent had to be filled in before starting the compilation of the questionnaire.

All participants were able to modify their given answer through designated buttons, but once the questionnaire was filled in and sent, the content could no longer be edited. All collected data was downloaded and stored on a password-protected computer and anonymized before being sent for statistical analysis, to maintain confidentiality and to perform a blind statistical analysis.

### Data analysis

The Survey Monkey® software allowed the authors to extrapolate the raw data and to export it into a Microsoft Excel spreadsheet.

The categorical demographic information was analysed by computing frequency and its percentage and it was used to define the features of the study population, the level of experience of the investigated professionals and the number of patients with shoulder arthroplasty treated in 1 year.

As for other previous surveys conducted in Italy [26, 39], a Chi Square (χ^2^) test or Fisher’s exact test (if cell size were below 5) were run to study differences among subgroups of the sample according to their clinical experience (years), academic background, the number of patients with shoulder arthroplasty treated in 1 year, and degree in OMPT (a PT’s specialization according to International Federation of Orthopaedic Manipulative Physical Therapists-IFOMPT training standards) [40]. In case of a significant χ^2^, adjusted standardized residuals [41] with their Bonferroni-corrected *p*-value were calculated in order to determinate which cells of contingency table contributed most to the significant effect [42, 43]. The data were analysed with SPSS Statistical Software (SPSS. Version 20 for Windows; Release 13.0.1. SPSS Inc., Chicago, IL; 2004). The *p*-value was set at α < 0.05.

## Results

### Sample

A total of 607 participants completed this survey (50.2%, *n* = 305/607, female; 49.8%, *n* = 302/607 male). The findings showed that more than half of the participants were under 35 years old (58.6%, *n* = 356/607) and had less than 10 years of clinical experience (55.9%, *n* = 339/607).

Regarding the number of patients with shoulder arthroplasty treated in a year, 61.9% (*n* = 376/607) of the participants stated that they treated less than 4 patients with shoulder arthroplasty during a year, while only 8.7% (*n* = 53/607) treated more than 12 patients per year.

The characteristics of the pool of participants, including their work setting and university qualification are reported in Table 1.

**Table 1:** Descriptive demographic information, overall sample

**Question**	**Multiple choice**	**Frequency (N)**	**Percentage (%)**	**C.I**
Gender (**Q1**)	Female	305	50.2	46.3–54.2
	Male	302	49.8	45.8–53.7
Age (**Q2**)	< 30	215	35.4	31.6–39.2
	30–35	141	23.2	19.9–26.6
	36–40	72	11.9	9.3–14.4
	41–45	51	8.4	6.2–10.6
	> 45	128	21.1	17.9–24.3
How many years have you been working as a physiotherapist? (**Q3**)	< 5	182	30.0	26.3–33.6
	5–10	157	25.9	22.4–29.3
	11–15	93	15.3	12.5–18.2
	16–20	51	8.4	6.2–10.6
	> 20	124	20.4	17.2–23.6
Prevalent work place (**Q4**)	Nursing home	23	3.8	2.3–5.3
	Sport team	6	1.0	0.2–1.8
	Public or private healthcare facility focused on orthopedic surgery	48	7.9	5.8–10.1
	Private healthcare facility NON focused on orthopedic surgery	259	42.7	38.7–46.6
	Public healthcare facility NON focused on orthopedic surgery	120	19.8	16.6–22.9
	Private outpatient clinic (self-employee)	151	24.9	21.4–28.3
Which is your highest academic qualification? (**Q5**)	Bachelor Degree	541	89.1	86.7–91.6
	Master of Science Degree	66	10.9	8.4–13.3
Did you obtain the university qualification for OMPT qualifying title? (**Q6**)	Yes	120	19.8	16.6–22.9
	No	487	80.2	77.1–83.4
How many patients with shoulder replacement (any type) did you visit in a year? (**Q7**)	< 4	376	61.9	58.1–65.8
	4–8	137	22.6	19.2–25.9
	9–12	41	6.8	4.8–8.7
	> 12	53	8.7	6.5–11.0

Acronyms: *C.I.* Confidence interval, *N* Number, *OMPT* Orthopaedic Manipulative Physical Therapist, *Q* Question

The subgroup of PTs with OMPT qualification were 19.8% (*n* = 120/607) of the whole pool recruited. Most of them (75.9%, *n* = 91/120) reported to be under 35 years old and to have less than 10 years of clinical experience (68.3%, *n* = 82/120). In addition, 65.0% (*n* = 78/120) of the OMPTs declared to treat less than 4 patients with shoulder arthroplasty per year, and 7.5% (*n* = 9/120) more than 12 patients per year.

The demographic data analysis of the OMPTs pool is shown in Table 2.

**Table 2:** Descriptive demographic information, OMPTs sample

**Question**	**Multiple choice**	**Frequency (N)**	**Percentage (%)**	**C.I**
Gender (**Q1**)	Female	53	44.2	40.2–48.1
	Male	67	55.8	51.9–59.8
Age (**Q2**)	< 30	50	41.7	37.7–45.6
	30–35	41	34.2	30.4–37.9
	36–40	16	13.3	10.6–16.0
	41–45	5	4.2	2.6–5.8
	> 45	8	6.7	4.7–8.6
How many years have you been working as a physiotherapist? (**Q3**)	< 5	33	27.5	23.9–31.1
	5–10	49	40.8	36.9–44.7
	11–15	22	18.3	15.3–21.4
	16–20	5	4.2	2.6–5.8
	> 20	11	9.2	6.9–11.5
Prevalent work place (**Q4**)	Nursing home	1	0.8	0.1–1.6
	Sport team	1	0.8	0.1–1.6
	Public or private healthcare facility focused on orthopedic surgery	8	6.7	4.7–8.6
	Private healthcare facility NON focused on orthopedic surgery	52	43.3	39.4–47.3
	Public healthcare facility NON focused on orthopedic surgery	18	15.0	12.2–17.8
	Private outpatient clinic (self-employee)	40	33.3	29.6–37.1
Which is your highest academic qualification? (**Q5**)	Bachelor degree	101	84.2	81.3–87.1
	Master of Science degree	19	15.8	12.9–18.7
How many patients with shoulder replacement (any type) did you visit in a year? (**Q7**)	< 4	78	65.0	61.2–68.8
	4–8	29	24.2	20.8–27.6
	9–12	4	3.3	1.9–4.8
	> 12	9	7.5	5.4–9.6

Acronyms: *C.I.* Confidence interval, *N* Number, *OMPT* Orthopaedic Manipulative Physical Therapist, *Q* Question

### Responses to the questionnaire

This survey investigated and analysed the Italian PTs clinical practice in the rehabilitation management of TSA and RTSA patients.

#### Section 1 – shoulder arthroplasty rehabilitation (general questions)

Patient education was thought to be very relevant for a good functional recovery by 88.5% (*n* = 537/607) of the participants. Only 7.0% (*n* = 4/607) of the participants considered it to be irrelevant (**Q8**).

Regarding the pain and swelling management in the early post-surgery stages of shoulder arthroplasty, 82.9% (*n* = 503/607) of PTs reported that they deliver a program based on the patient’s education, ice application, cautious passive joint mobilization and gentle active joint exercises (**Q9**).

Sixty-six point six percent of participants (*n* = 404/607) declared to primarily use manual therapy and therapeutic exercise with progressive load, while only 5.1% (*n* = 31/607) and 0.2% (*n* = 1/607) of PTs claimed to adopt aquatic therapy or physical modalities (i.e., laser therapy, diathermy), respectively (**Q10**).

Almost half of the participants (49.9%; *n* = 303/607) did not administer patient-reported outcome measures (**Q11**). Conversely, a large proportion of the sample (83.9%; *n* = 509/607) used strength measurement, active ROM (aROM) and passive ROM (pROM) as non-patient-reported outcome measures (**Q12**).

The detailed answers to general questions for shoulder rehabilitation are shown in Table 3 (**Q8-Q12**).

**Table 3:** Data analysis, overall (Sections 1, 2, 3)

**Question**	**Multiple choice**	**Frequency (N)**	**Percentage (%)**	**C.I**
**Section 1 | Shoulder replacement rehabilitation -general question-**				
How important do you think patient education is for a good functional recovery after shoulder replacement? (**Q8**)	I do not know	5	0.8	0.1–1.5
	Not very important	4	0.7	0.0–1.3
	Relatively important	61	10.0	7.7–12.4
	Very important	537	88.5	85.9–91.0
In your clinical practice, how do you manage pain and swelling in the immediate post-operative period after shoulder replacement (0–3 weeks)? (**Q9**)	Cautious passive joint mobilization and introduction of gentle active joint exercises	15	2.5	1.2–3.7
	Patient education and cautious passive joint mobilization	64	10.5	8.1–13.0
	Patient education, ice, treatment of oedema, cautious passive joint mobilization and introduction of gentle active joint exercises	503	82.9	79.9–85.9
	Patient education and treatment of oedema	25	4.1	2.5–5.7
In your clinical practice, which treatment strategies do you mainly prefer during rehabilitation of a patient with TSA? (**Q10**)	Aquatic therapy	31	5.1	3.4–6.9
	Modalities (e.g., electrotherapy, laser therapy, diathermy)	1	0.2	-0.2–0.5
	Manual therapy and therapeutic exercise with progressive load	404	66.6	62.8–70.3
	Manual therapy and therapeutic exercise with progressive load, modalities	171	28.2	24.6–31.7
In your clinical practice, do you use self-reported outcome measures (self-assessment questionnaires that are filled indirectly by the patient, e.g., DASH) at the beginning and/or end of the rehabilitation treatment after shoulder replacement? (**Q11**)	No	303	49.9	45.9–53.9
	I don’t know any	27	4.4	2.8–6.1
	Yes, sometimes	195	32.1	28.4–35.8
	Yes, always	82	13.5	10.8–16.2
In your clinical practice, which non-self-reported outcome measures (measures for which the operator observes a certain variable and assigns a score, e.g., ROM) do you mainly use to record the obtained results from the treatment of patients after shoulder replacement? (**Q12**)	Strength assessment	3	0.5	-0.1–1.1
	aROM assessment	69	11.4	8.8–13.9
	pROM assessment	26	4.3	2.7–5.9
	All previous answers	509	83.9	80.9–86.8
**Section 2 | Rehabilitation after TSA**				
Which movement is important to avoid, as to prevent TSA dislocation? (**Q13**)	Shoulder abduction and external rotation	264	43.5	39.5–47.4
	Shoulder adduction and internal rotation	87	14.3	11.5–17.1
	End-range shoulder anterior flexion	21	3.5	2.0–4.9
	Shoulder internal rotation, adduction and extension	235	38.7	34.8–42.6
In your clinical practice, how do you manage pROM recovery in patients with TSA? (**Q14**)	pROM from 4th up to 8th week with shoulder forward flexion and external rotation movements	25	4.1	2.5–5.7
	pROM from 8^th^ up to 12^th^ week with progression, according to patient’s tolerance	148	24.4	21–27.8
	pROM up to first 6 weeks, with shoulder forward flexion, abduction, internal rotation and 15°-30° limit of external rotation; full pROM in all direction of movements from 6 to 12^th^ week	377	62.1	58.3–66
	pROM up to first 6 weeks, in all direction of movements; full pROM at 6^th^ week	57	9.4	7.1–11.7
In your clinical practice, how do you manage aROM recovery in patients with TSA? (**Q15**)	aROM < 90° of shoulder forward flexion and abduction at 3–4 weeks; aROM > 90° from 6 to 12^th^ weeks;full aROM at 3 months	268	44.2	40.2–48.1
	assisted aROM with pulley up to first 4 weeks; aROM of shoulder forward flexion from 4 to 8^th^ week; full aROM in all directions of movement with tolerance from 8 to 12^th^ week	147	24.2	20.8–27.6
	assisted aROM for 6 weeks; full aROM in all directions of movement at 9 weeks	44	7.2	5.2–9.3
	assisted aROM for 6 weeks; full aROM at 3 months	148	24.4	21–27.8
In your clinical practice, when do you introduce isometric exercise in patients with TSA? (**Q16**)	0–3 weeks	275	45.3	41.3–49.3
	4–6 weeks; isometric contraction of scapular muscles and distal forearm muscles; isometric contraction in internal and external rotation from 6 to 10^th^ week	188	31.0	27.3–34.7
	4–6 weeks	123	20.3	17.1–23.5
	5–10 weeks	21	3.5	2.0–4.9
In your clinical practice, when do you think it is necessary to start with progressive muscle strengthening in patients with TSA? (**Q17**)	6–8 weeks	220	36.2	32.4–40.1
	9–12 weeks	105	17.3	14.3–20.3
	Over 12 weeks	23	3.8	2.3–5.3
	According to patient’s joint recovery	259	42.7	38.7–46.6
Which of these muscles are a priority during strengthening phase in patients with TSA? (**Q18**)	Scapular muscles	11	1.8	0.8–2.9
	Scapular muscles and rotator cuff muscles	113	18.6	15.5–21.7
	Scapular muscles and deltoid	84	13.8	11.1–16.6
	Scapular muscles and rotator cuff muscles, deltoid, biceps, triceps	399	65.7	62–69.5
What is the most common complication that can occur following TSA surgery? (**Q19**)	Infection	63	10.4	8–12.8
	Instability of the glenoid prosthetic component	201	33.1	29.4–36.9
	Failure of the subscapularis tendon	153	25.2	21.8–28.7
	Dislocation	190	31.3	27.6–35.0
In your clinical practice, following TSA surgery, when should the patient be instructed to mainly return to ADLs (e.g. washing, dressing, combing their hair, cooking) (**Q20**)	6–9 weeks	55	9.1	6.8–11.3
	9–12 weeks	71	11.7	9.1–14.3
	> 12 weeks	32	5.3	3.5–7.0
	From week 6 onwards, depending on the patient’s recovery (pain reduction, ROM recovery) and the specificity of each activity^a^	449	74.0	70.5–77.5
In your clinical practice, following TSA surgery, when do you expect the patient to be able to return to sport? (in details: involving the upper limb, non-contact sport and non-throwing sport) (**Q21**)	6–12 weeks	19	3.1	1.7–4.5
	13–24 weeks	167	27.5	24.0–31.1
	7 months–1 year	340	56.0	52.1–60.0
	Over a year	81	13.3	10.6–16.0
**Section 3 | Rehabilitation after RTSA**				
Which movement is important to avoid, as to prevent RTSA dislocation? (**Q22**)	Shoulder abduction and external rotation	182	30.0	26.3–33.6
	Shoulder abduction and internal rotation	65	10.7	8.2–13.2
	End-range shoulder anterior flexion	35	5.8	3.9–7.6
	Shoulder internal rotation, adduction and extension	325	53.5	49.6–57.5
In your clinical practice, how do you manage pROM recovery in patients with RTSA? (**Q23**)	pROM up to first 6 weeks, with 90°-120° shoulder forward flexion and till 30°external rotation with tolerance; full pROM from 6 to 12^th^ week	300	49.4	45.4–53.4
	pROM from 8^th^ up to 12^th^ week with progression, depending on patient’s tolerance	116	19.1	16.0–22.2
	pROM up to first 6 weeks; full pROM from 6 to 12^th^ week, included full shoulder external rotation	128	21.1	17.8–24.3
	No pROM in the first 6 weeks; pROM shoulder movements with tolerance after 6 weeks	63	10.4	8.0–12.8
In your clinical practice, how do you manage aROM recovery in patients with RTSA? (**Q24**)	aROM till 90°shoulder forward flexion and 30° external rotation in the first 6 weeks; aROM till 90° of shoulder forward flexion till 12^th^ week; full aROM with tolerance from 12 to 16^th^ week	216	35.6	31.8–39.4
	aROM till 120° shoulder forward flexion and 30° external rotation in the first 6 weeks; full aROM over 6 weeks	30	4.9	3.2–6.7
	aROM in all direction of movements with tolerance from 6^th^ week; full aROM from 12 to 16^th^ week	119	19.6	16.4–22.8
	Hand, wrist and elbow aROM maintenance up to first 6 weeks; full aROM from 12 to 16^th^ week with progression, according to patient’s tolerance	242	39.9	36.0–43.8
In your clinical practice, when do you introduce isometric exercise in patients with TSA? (**Q25**)	0–3 weeks	249	41.0	37.1–44.9
	4–6 week	159	26.2	22.7–29.7
	4–6 weeks; isometric contraction of scapular muscles and distal forearm muscles; isometric contraction in internal and external rotation from 6 to 10^th^ week	163	26.9	23.3–30.4
	> 6 weeks	36	5.9	4.1–7.8
In your clinical practice, when do you think it is necessary to start with progressive muscle strengthening in patients with RTSA? (**Q26**)	6–8 weeks	222	36.6	32.7–40.4
	9–12 weeks	119	19.6	16.4–22.8
	Over 12 weeks	48	7.9	5.8–10.1
	According to patient’s joint recovery	218	35.9	32.1–39.7
Which of these muscles are a priority during strengthening phase in patients with RTSA? (**Q27**)	Deltoid	77	12.7	10–15.3
	Rotator cuff muscles	85	14.0	11.2–16.8
	Scapular muscles	32	5.3	3.5–7.0
	Deltoid and scapular muscles	413	68.0	64.3–71.7
What is the most common complication that can occur following RTSA surgery? (**Q28**)	Scapular notch erosion	258	42.5	38.6–46.4
	Acromial fracture	76	12.5	9.9–15.2
	Infection	56	9.2	6.9–11.5
	Dislocation	217	35.7	31.9–39.6
In your clinical practice, following RTSA surgery, when should the patient be instructed to mainly return to ADLs (e.g. washing, dressing, combing their hair, cooking) (**Q29**)	6–9 weeks	65	10.7	8.2–13.2
	9–12 weeks	90	14.8	12–17.7
	> 12 weeks	52	8.6	6.3–10.8
	From week 6 onwards, depending on the patient’s recovery (pain reduction, ROM recovery) and the specificity of each activity^a^	400	65.9	62.1–69.7
In your clinical practice, following RTSA surgery, when do you expect the patient to be able to return to sport? (in details: involving the upper limb, non-contact sport and non-throwing sport) (**Q30**)	6–12 weeks	24	4.0	2.4–5.5
	13–24 weeks	119	19.6	16.4–22.8
	7 months–1 year	283	46.6	42.7–50.6
	Over a year	181	29.8	26.2–33.5

Acronyms: *ADLs* Activities of daily living, *aROM* Active range of motion, *C.I.* Confidence interval, *DASH* Disability of the Arm, Shoulder and Hand Questionnaire, *N* Number, *pROM* Passive range of motion, *ROM* Range of motion, *Q* Question, *RTSA* Reverse Total Shoulder Arthroplasty, *TSA* Total Shoulder Arthroplasty

^a^From the simplest movement as “washing the face” to the most complex one as “wearing a coat or reaching targeted overhead movement”

#### Section 2 – TSA rehabilitation

The specific movement aimed at avoiding TSA dislocation is abduction associated with external rotation [44] and 43.5% (*n* = 264/607) of the interviewed PTs answered correctly (**Q13**).

In order to recover pROM, 62.1% (*n* = 377/607) of the participants work in order to gain anterior flexion, abduction, internal rotation, external rotation until 30°, with full pROM in all directions granted at 6–12 weeks (**Q14**).

Regarding the aROM (**Q15**), 44.2% (*n* = 268/607) of the participants claimed to use active-assisted procedures within a range under 90° of elevation and abduction at 3–4 weeks and more than 90° at 6–12 weeks, with full recovery at 3 months.

Almost half of the sample (45.3%; *n* = 275/607) reported that they introduce isometric exercises (**Q16**) in the first 3 weeks, while 31.0% (*n* = 188/607) of the PTs claimed to introduce isometric exercises at 4–6 weeks for the scapular muscles and at 6–10 weeks for the internal and external rotator cuff muscles.

When asked about progressive muscle strengthening (**Q17**), 42.7% of participants (*n* = 259/607) stated that they normally begin this stage of rehabilitation program depending on the patient’s actual stage of recovery. Regarding the specific muscle strengthening (**Q18**), 65.7% (*n* = 399/607) of PTs believed that they should aim at strengthen scapular and rotator cuff muscles, deltoid, biceps and triceps.

The instability of the glenoid prosthetic component was identified as the most frequent complication in patients with TSA (31.1%, *n* = 201/607) [15], followed by its dislocation (31.3%; *n* = 190/607) and infection (10.4%; *n* = 63/607) (**Q19**).

Most participants reported a minimum cut-off for return to activities of daily living (ADLs) of 6 weeks (74.0%; *n* = 449/607) (**Q20**) and indicated a period of 7 months to 1 year for return to sport (RTS) (56.0%; *n* = 340/607) (**Q21**).

The detailed answers to TSA rehabilitation questions for shoulder rehabilitation are reported in Table 3 (**Q13-Q21**).

#### Section 3 - RTSA rehabilitation

The specific movement aimed at avoiding the RTSA dislocation is the internal rotation, in conjunction to adduction and extension [9, 15, 45], and 53.5% (*n* = 325/607) of participants responded correctly (**Q22**).

In order to recover pROM, 49.4% (*n* = 300/607) of the participants work for the first 6 weeks to recover anterior flexion from 90° to 120° and external rotation until 30°, with progression towards full anterior flexion and external rotation at 6–12 weeks and full pROM in all directions granted at 12–16 weeks (**Q23**).

Regarding aROM (**Q24**), 39.9% (*n* = 242/607) of the participants stated to achieve complete active recovery toward progression from 6 to 12 weeks according to patient’s threshold tolerance (**Q24**).

Less than half of the sample (42.0%; *n* = 249/607) declared to introduce isometric exercises (**Q25**) in the first 3 weeks.

When asked about progressive muscle strengthening (**Q26**), 36.6% of participants (*n* = 222/607) stated that they begin this stage of rehabilitation after 6 to 8 weeks from surgery, while other 35.9% (*n* = 218/607) declared to rely on patient’s actual stage of recovery. Regarding the focus on specific muscle strengthening (**Q27**), 68.0% (*n* = 413/607) of PTs believed that scapular muscles and the deltoid muscle should be strengthened.

Thirty-five point seven (*n* = 217/607) of respondents identified that the most frequent complication in patients with RTSA is shoulder prosthesis dislocation [15], followed by scapular notching (42.5%; *n* = 258/607) and infection (9.2%; *n* = 56/607) (**Q28**).

Finally, most participants reported a minimum cut-off for return to ADLs of 6 weeks (65.9%; *n* = 400/607) (**Q29**) and indicated a period of 7 months to 1 year for RTS (46.6%; *n* = 283/607) (**Q30**).

The detailed answers to the questions about RTSA rehabilitation for shoulder rehabilitation are reported in Table 3 (**Q22-Q30**).

### Subgroup analyses

This survey also assessed the association of the participants’ a) years of clinical experience; b) highest academic qualification c) degree in OMPT and d) number of patients with shoulder arthroplasty treated in 1 year, comparing their answers to six questions (**Q13, Q18, Q19, Q22, Q27, Q28**) to the current recommendations (Tables 4, 5, 6 and 7).

**Table 4:** Inference between answers to questions Q13, Q18, Q19, Q22, Q27, Q28 and year of clinical experience. The percentages underlined in each catagory mean the percentage of clinicians that correctly answered the questions

**Question**	How many years have you been working as a physiotherapist? (**Q3**)					
	** < 5**	**5–10**	**11–15**	**16–20**	** > 20**	*P*-value**
Which movement is important to avoid, as to prevent TSA dislocation? *Correct answer: shoulder abduction and external rotation* (**Q13**)	44.0%	49.7%	35.5%	39.2%	42.7%	0.258
Which of these muscles are a priority during strengthening phase in patients with TSA? *Correct answer: scapular muscles and rotator cuff muscles* (**Q18**)	21.4%	19.1%	14.0%	15.7%	18.5%	0.630
What is the most common complication that can occur following TSA surgery? *Correct answer: instability of the glenoid prosthetic component* (**Q19**)	34.1%	29.3%	30.1%	47.1%	33.1%	0.202
Which movement is important to avoid, as to prevent RTSA dislocation? *Correct answer: shoulder internal rotation, adduction and extension* (**Q22**)	61.5%	49.7%	62.4%	52.9%	40.3%	0.002*
**Adjusted residual**	2.58	-1.12	1.85	-0.08	-3.30	
*Residual’s p-values (Bonferroni p-values* = *0.0050)*	0.009738	0.259986	0.063731	0.928373	**0.000937**	
Which of these muscles are a priority during strengthening stage phase in patients with RTSA? *Correct answer: deltoid and scapular muscles* (**Q27**)	65.9%	70.1%	79.6%	72.5%	58.1%	0.014*
**Adjusted residual**	-0.72	0.63	2.59	0.72	-2.67	
*Residual’s p-values (Bonferroni p-values* = *0.0050)*	0.466647	0.527598	0.009562	0.470555	0.007578	
What is the most common complication that can occur following RTSA surgery? *Correct answer: dislocation* (**Q28**)	35.7%	33.1%	31.2%	47.1%	37.9%	0.354

Acronyms: *Q* Question, *RTSA* Reverse Total Shoulder Arthroplasty, *TSA* Total Shoulder Arthroplasty

^*^Significant *p*-value; statistically significant differences according to the corrected residuals are shown in bold

^**^P-value referred to chi squared test

**Table 5:** Inference between answers to questions Q13, Q18, Q19, Q22, Q27, Q28 and highest academic qualification. The percentages underlined in each catagory mean the percentage of clinicians that correctly answered the questions

**Question**	Which is your highest academic qualification? (**Q5**)		
	**Bachelor’s Degree**	**Master’s Degree**	*P*-value**
Which movement is important to avoid, as to prevent TSA dislocation? *Correct answer: shoulder abduction and external rotation* (**Q13**)	44.4%	36.4%	0.216
Which of these muscles are a priority during strengthening phase in patients with TSA? *Correct answer: scapular muscles and rotator cuff muscles* (**Q18**)	19.4%	12.1%	0.151
What is the most common complication that can occur following TSA surgery? *Correct answer: instability of the glenoid prosthetic component* (**Q19**)	33.1%	33.3%	0.968
Which movement is important to avoid, as to prevent RTSA dislocation? *Correct answer: shoulder internal rotation, adduction and extension* (**Q22**)	54.0%	50.0%	0.541
Which of these muscles are a priority during strengthening phase in patients with RTSA? *Correct answer: deltoid and scapular muscles* (**Q27**)	68.6%	63.6%	0.416
What is the most common complication that can occur following RTSA surgery? *Correct answer: dislocation* (**Q28**)	35.1%	40.9%	0.354

Acronyms: *Q* Question, *RTSA* Reverse Total Shoulder Arthroplasty, *TSA* Total Shoulder Arthroplasty

^**^P-value referred to chi squared test

**Table 6:** Inference between answers to questions Q13, Q18, Q19, Q22, Q27, Q28 and OMPT qualification. The percentages underlined in each catagory mean the percentage of clinicians that correctly answered the questions

**Question**	Did you obtain the university qualification for OMPT qualifying title? (**Q6**)		
	**Yes**	**No**	*P*-value**
Which movement is important to avoid, as to prevent TSA dislocation? *Correct answer: shoulder abduction and external rotation* (**Q13**)	50.8%	41.7%	0.070
Which of these muscles are a priority during strengthening phase in patients with TSA? *Correct answer: scapular muscles and rotator cuff muscles* (**Q18**)	20.0%	18.3%	0.664
What is the most common complication that can occur following TSA surgery? *Correct answer: instability of the glenoid prosthetic component* (**Q19**)	36.7%	32.2%	0.356
Which movement is important to avoid, as to prevent RTSA dislocation? *Correct answer: shoulder internal rotation, adduction and extension* (**Q22**)	67.5%	50.1%	0.001*
**Adjusted residual**	3.42	-3.42	
*Residual’s p-values (Bonferroni p-values* = *0.0050)*	**0.000620**	**0.000620**	
Which of these muscles are a priority during strengthening phase in patients with RTSA? *Correct answer: deltoid and scapular muscles* (**Q27**)	76.7%	65.9%	0.024*
**Adjusted residual**	2.26	-2.26	
*Residual’s p-values (Bonferroni p-values* = *0.0050)*	0.023663	0.023663	
What is the most common complication that can occur following RTSA surgery? *Correct answer: dislocation* (**Q28**)	27.5%	37.8%	0.035*
**Adjusted residual**	-2.10	2.10	
*Residual’s p-values (Bonferroni p-values* = *0,0050)*	0.035279	0.035279	

Acronyms: *OMPT* Orthopaedic Manipulative Physical Therapist, *Q* Question, *RTSA* Reverse Total Shoulder Arthroplasty, *TSA* Total Shoulder Arthroplasty

^*^Significant *p*-value; statistically significant differences according to the corrected residuals are shown in bold

^**^P-value referred to chi squared test

**Table 7:** Inference between answers to questions Q13, Q18, Q19, Q22, Q27, Q28 and number of patients with shoulder replacement treated in one year. The percentages underlined in each catagory mean the percentage of clinicians that correctly answered the questions

**Question**	How many patients with shoulder replacement (any type) did you visit in one year? (**Q7**)				
	** < 4**	**4–8**	**9–12**	** > 12**	*P*-value**
Which movement is important to avoid, as to prevent TSA dislocation? *Correct answer: shoulder abduction and external rotation* (**Q13**)	44.7%	38.0%	48.8%	45.3%	0.483
Which of these muscles are a priority during strengthening phase in patients with TSA? *Correct answer: scapular muscles and rotator cuff muscles* (**Q18**)	19.1%	17.5%	17.1%	18.9%	0.970
What is the most common complication that can occur following TSA surgery? *Correct answer: instability of the glenoid prosthetic component* (**Q19**)	31.9%	32.8%	29.3%	45.3%	0.254
Which movement is important to avoid, as to prevent RTSA dislocation? *Correct answer: shoulder internal rotation, adduction and extension* (**Q22**)	54.0%	49.6%	48.8%	64.2%	0.303
Which of these muscles are a priority during strengthening phase in patients with RTSA? *Correct answer: deltoid and scapular muscles* (**Q27**)	67.6%	70.1%	78.0%	58.5%	0.220
What is the most common complication that can occur following RTSA surgery? *Correct answer: dislocation* (**Q28**)	37.8%	28.5%	39.0%	37.7%	0.250

Acronyms: *Q* Question, *RTSA* Reverse Total Shoulder Arthroplasty, *TSA* Total Shoulder Arthroplasty

^**^P-value referred to chi squared test

Table 4 shows the association between the six correct answers and the years of clinical experience, the differences between the different groups were reported only for **Q22** (*p* = 0.002), namely the question which investigated the importance to avoid specific movement in order to prevent the RTSA dislocation. According to Bonferroni’s post-hoc analysis, participants with more than 20 years of experience were more likely to report the correct movement to be avoided to prevent RTSA dislocation (adjusted residual = -3.30, residual *p*-value = 0.000937, Bonferroni-corrected *p*-value = 0.0050).

The association between **Q13, Q18, Q19, Q22, Q27, Q28** the answers and the highest academic degree stated by PTs is reported in Table 5; the results showed no significant differences. On the other hand, PTs with OMPT qualification were more likely to report the movements to be avoided to prevent RTSA dislocation (**Q22**) (adjusted residual = 3.42, residual *p*-value = 0.000620, Bonferroni-corrected *p*-value = 0.0050). However, as for RTSA, in **Q27** about the knowledge of the priority of muscle progressive strengthening, and **Q28**, about the most common complications, Bonferroni’s post-hoc analysis did not show any significance (Table 6).

Finally, no statistically significant difference was reported using chi-square test, with regards to the association between the answers to **Q13, Q18, Q19, Q22, Q27, Q28** and the number of patients with shoulder arthroplasty treated in 1 year (Table 7).

## Discussion

The primary purpose of this survey was to investigate and analyse the Italian PTs clinical practice in the rehabilitation management of patients with TSA and RTSA. The overall results showed that Italian PTs mainly employ manual therapy and therapeutic exercise with progressive load as their main strategies in the treatment of patients with shoulder arthroplasty. Moreover, patient education strategies are considered as important in order to guarantee a good functional recovery. Regarding another fundamental aspect of clinical practice, Italian PTs are more keen to apply non self-reported outcome measures (e.g., ROM and strength assessment), compared to self-reported outcome measures (e.g., Disability of the Arm, Shoulder and Hand (DASH) Questionnaire), although the use of the latter is highly recommended by the literature [46, 47].

### TSA

As far as the rehabilitation management of patients with TSA is concerned, the analysis of the collected data showed that the most of Italian PTs favourably opted for early joint mobilization (pROM restoration since the first week). The early mobilization choice probably aims at gaining a faster functional independence and at avoiding post-operative stiffness - often described as the main complication and reason for dissatisfaction in patients who had a TSA surgery performed [48, 49]. To date, only one randomized controlled trial compared early rehabilitation treatment (starting from the first post-operative day) to delayed rehabilitation treatment (starting 4 weeks after surgery) in patients with TSA [34]: the results showed that participants who were randomized to early rehabilitation reported a faster recovery of ADL independence, but at 1 year post-surgery follow-up no statistically nor clinically relevant differences between the two groups were to be found [34]. Furthermore, no data on joint post-surgery stiffness with delayed rehabilitation have been reported. Although post-surgical stiffness is considered a strong factor in PTs’ treatment program choices, there is currently insufficient consensus on early or delayed mobilization after TSA [7].

The results of this survey also highlighted that Italian PTs use flexion, abduction, internal rotation, limited external rotation to 30° pROM as rehabilitation strategies during the first 6 weeks post-surgery to promote the tissue restoration. Most participants stated that these treatment strategies are adopted for a complete pROM recovery in all directions of movement between the 6^th^ and the 12^th^ week after TSA, while a full aROM is expected to be regained in 3 months. These rehabilitation strategies agree with the Consensus Statement on rehabilitation after TSA conducted by the American Society of Shoulder and Elbow Therapists (ASSET), published in 2020 [47].

In addition, regarding the muscle strengthening phase for TSA patients, most Italian PTs focused predominantly on scapular and rotator cuff muscles, deltoid, biceps and triceps. Their choice is supported by several studies concerning the appropriate strategies for a good functional recovery [50, 51], the correct restoration of glenohumeral kinematics, the reduction of joint and implant stress levels [20, 51], and the improvement of function and ADLs in shoulder arthroplasty [52]. Moreover, strength exercises of the RC muscles may help to support the prostheses durability [51], limiting possible cranial shifts of the humeral component and the subsequent instability of the whole implant [31].

On the other hand, this survey participants had discordant opinions about the correct timing to start the strengthening phase: almost half of the participants were not able to assign a precise timing: in fact, many PTs concluded that shoulder strengthening in TSA patients should start according to the patient’s clinical status. Similarly, the current evidence in the literature is not supported by any study of high methodological quality indicating the best time to start the strengthening phase and its progression [7].

Most participants indicated that patients should return to practice their sport activities after TSA between 7 and 12 months post-surgery. Once again, evidence of reliable data is currently still missing, being strongly influenced by a lot of variable indications from the surgical point of view [53, 54]. However, according to the American PTs’ opinion, the ASSET consensus statement reported that it is possible to introduce sport-specific exercises from the 4^th^ post-operative month, without any joint restriction and respecting the patient’s tolerance, goals, needs, and preferences [47]. Nevertheless, a clear cut-off time for RTS is not indicated.

### RTSA

The present survey revealed a considerable heterogeneity regarding the rehabilitation management of patients with RTSA. Specifically, variable indications arose about pROM and aROM recovery strategies. The patient’s tolerance seems to be used as main criterion for progression by Italian PTs: an already well-known lack of supporting evidence has been highlighted, regarding pROM early [51, 55–58] or delayed [59–61] mobilization and relative timing of aROM mobilization strategies introduction [51]. Notably, the start of pROM and aROM recovery indication are often depending on surgeons’ prescriptions, and therefore present a very high variability.

Moreover, this survey pool of PTs showed similar results for strengthening phase in their RTSA patients. Similarly to the literature current evidence [51], no clear agreement has been reached upon the right time to introduce the strengthening phase.

Conversely, a significant agreement between the current evidence [51] and the data extrapolated from this survey could be highlighted regarding target muscles to strengthen frequently: most Italian PTs focused predominantly on scapular muscle and deltoid muscle during their strengthening phase in RTSA patients. Actually, due to the biomechanical complexity of RTSA, the joint rotation centre translates inferiorly and medially (in relation to the glenoid convex surface), with significant increase in the deltoid lever arm [9]. Consequently, deltoid strengthening, together with scapular muscles, is fundamental in order to gain functional and effective capacity [50, 62].

Finally, regarding the RTS issue, the survey showed that almost all the participants believe that patients should be able to go back practicing their sport activities, after RTSA implantation, between the 7 and 12 months post-surgery. Unlike what was previously described for TSA, this Italian PTs practice meets surgeons’ typical indications/recommendations – which tend indeed to be more restrictive than those usually adopted for anatomical arthroplasty [54, 63, 64]. However, even for patients with RTSA implantation, no literature consensus has been actually achieved and a clear cut-off time for RTS is not indicated [8, 54, 65].

A recent systematic review described and summarized the current available protocols for post-TSA and RTSA rehabilitation [22], highlighting poor methodological quality indications/prescriptions and, on the other hand, an extensive reliance on expert opinion [22]. Furthermore, another systematic review evaluated the efficacy of post-surgical rehabilitation in TSA or RTSA patients, including only randomized controlled trials [1]. Unfortunately, only one eligible study could be found [34] and the authors therefore emphasized the urgent need for other good quality randomized controlled trials in order to identify the best practice in TSA and RTSA patients’ management [1].

In order to provide the best possible outcomes, PTs must consider of all factors that could influence their patients’ prognosis, such as their adherence to the rehabilitation program [18, 19], their previous pathology severity [5, 10, 12, 18], their pre-operative shoulder function [5, 9, 12], and their general health status, age and gender [12]. Furthermore, in order to propose an adequate rehabilitation plan, PTs should be aware of the surgical techniques and types of implants used. These factors represent two independent prognostic elements, which can highly influence the patient’s recovery [5]. For this reason, a multidisciplinary approach, together with an effective communication and collaboration with surgeons represents optimal musculoskeletal care and has a fundamental importance in creating appropriate treatments [9, 12, 21, 47] and to achieve the best possible results.

### Associations

As a cross sectional study, this survey also conducted an analysis of any existing association between current shoulder arthroplasty shared recommendations - regarding movements to avoid in order to prevent shoulder implant dislocation, important muscles to strengthen, and TSA and RTSA most frequent complications - and the following four independent variables: a) years of clinical experience; b) highest academic qualification c) OMPT qualification d) number of patients with shoulder arthroplasty treated in 1 year. These association revealed that participants with more than 20 years of experience and PTs with OMPT qualification were more likely to report the correct movement to avoid to prevent RTSA dislocation. No other association was found between other different respondents subgroups and evidence recommendations.

The results suggested that Italian PTs with OMPT specialization tend to be more adherent to the current evidence. As already speculated in other surveys conducted in Italy [26, 27, 66] it can be assumed that the OMPT academic specialization course can lead the professional path of PTs towards a broad understanding and application of indications, suggestions and practice which are solidly supported by current scientific evidence; however, this study being a cross-sectional survey, more prospective studies should be structured, aiming to support this assumption.

### Strength and limitations

As far as this study limitations are concerned, it is evident that this survey was distributed through different communication channels, predominantly linked to social networks. Therefore, the pool of the recruited participants could be represented only by those who are familiar with electronic devices and social media [67]. However, as already observed in other surveys within the musculoskeletal field [68], the choice of dissemination channels could be interpreted as a potential selection bias; on the other hand, it is also true that the use of electronic devices and social media is rather widespread among the health professionals community [69].

Secondly, this survey succeeded in recruiting a rather small number of PTs, especially those with a OMPT specialization. In particular, their sample accounts for a minority of the entire national OMPTs scenario - negatively affecting the transferability of the results.

On the other hand, the robust adherence to guidelines and reporting during the design of this study and while drafting the questionnaire, together with a careful statistical analysis and the use sample sub-divisions through independent variables - which allowed for a more detailed analysis - can be considered the main strengths of this study.

Finally, this study joins other 3 recent surveys conducted in Italy investigating the shoulder joint [26, 27, 66], and it represents the first real attempt to analyse the management of the rehabilitation path adopted by Italian PTs in managing patients with TSA and RTSA.

### Future perspectives

This survey represents a starting point for further studies aimed at monitoring the clinical practice of PTs in the rehabilitation management of TSA and RTSA patients in Italy and, optimistically, also in other countries. Being PTs themselves, the authors expect and hope that timely and careful observation of PTs’ habits and attitude towards patients with TSA and RTSA, as well as grounded and robust evidence on the effectiveness of the rehabilitation management, will be highlighted by researchers in the near future.

## Conclusions

The clinical practice of Italian PTs in the rehabilitation of patients with TSA and RTSA effectively reflects the indications of the literature as far as the strengthening of the main muscle groups and the prevention of movements which may result in a dislocation are concerned. Conversely, there are still substantial differences when considering the passive and active joint mobilization starting time and progression, muscle strengthening techniques (timing and dosage) and the return to sport management (timing). These disagreement aspects actually reflect the uncertainty of current literature degree of knowledge on post-surgical rehabilitation for shoulder prosthesis in the rehabilitation field.

## Supplementary Information

**Additional file 1.** Information letter.

**Additional file 2.** Questionnaire.

## Data Availability

All data generated or analysed during this study are included in this published article [and its supplementary information files].

## Abbreviations

TSA: Total Shoulder Arthroplasty
RTSA: Reverse Total Shoulder Arthroplasty
ROM: Range of Movement
RC: Rotator Cuff
PT: Physiotherapist
OMPT: Orthopaedic Manipulative Physical Therapist
aROM: Active Range of Movement
pROM: Passive Range of Movement
