# Supplementary material for: Rehabilitation following shoulder arthroplasty: a survey of current clinical practice patterns of Italian physiotherapists
Source: Arch Physiother. 2023 Jun 5;13:12. doi: 10.1186/s40945-023-00166-5 (PMC10243052; doi:10.1186/s40945-023-00166-5)
Supplement: Supplementary file 1 — Additional file 1. Information letter. [file 40945_2023_166_MOESM1_ESM.docx]

**Additional file 1 - Information letter**

Gentile collega Fisioterapista,

sono Michele Usai, studente del Master in Fisioterapia Muscolo-Scheletrica e Reumatologica dell’Università di Roma Tor Vergata.

Insieme ai colleghi Fabrizio Brindisino, Mariangela Lorusso, Sharon Marruganti e Mattia Salomon, sto conducendo un’indagine volta ad approfondire le conoscenze circa la pratica dei fisioterapisti italiani in tema di riabilitazione post-chirurgica in pazienti sottoposti ad artroprotesi di spalla.

Ti prego di dedicare qualche minuto del Tuo tempo rispondendo alle seguenti domande in base alla Tua esperienza personale ed indicare ciò che applichi quotidianamente nella Tua pratica clinica.

Le risposte sono completamente anonime e daranno un prezioso contributo al nostro lavoro di ricerca.

Grazie per la tua partecipazione.

Se accetti di partecipare volontariamente al questionario, clicca su “OK”.
