# Supplementary material for: Rehabilitation following shoulder arthroplasty: a survey of current clinical practice patterns of Italian physiotherapists
Source: Arch Physiother. 2023 Jun 5;13:12. doi: 10.1186/s40945-023-00166-5 (PMC10243052; doi:10.1186/s40945-023-00166-5)
Supplement: Supplementary file 2 — Additional file 2. Questionnaire. [file 40945_2023_166_MOESM2_ESM.docx]

**Additional file 2**

**Question 1** – Gender:

- Female
- Male

**Question 2** – Age:

- <30
- 30-35
- 36-40
- 41-45
- >45

**Question 3** – How many years have you been working as a physiotherapist?

- <5
- 5-10
- 11-15
- 16-20
- >20

**Question 4** – Prevalent work place:

- Nursing home
- Sport team
- Public or private healthcare facility (Focused on orthopedic surgery)
- Private healthcare facility
- Public healthcare facility
- Private outpatient clinic (self-employee)

**Question 5** – Which is your highest academic qualification?

- Bachelor Degree
- Master of Science Degree

**Question 6** – Did you obtain the university qualification for OMPT qualifying title?

- Yes
- No

**Question 7** – How many patients with shoulder replacement (any type) did you visit in a year?

- **<**4
- 4-8
- 9-12
- >12

**SECTION 1 | SHOULDER REPLACEMENT REHABILITATION -GENERAL QUESTION-**

**Question 8** – How important do you think patient education is for a good functional recovery after shoulder replacement?

- I do not know
- Not very important
- Relatively important
- Very important

**Question 9** – In your clinical practice, how do you manage pain and swelling in the immediate post-operative period after shoulder replacement (0-3 weeks)?

- Cautious passive joint mobilization and introduction of gentle active joint exercises
- Patient education and cautious passive joint mobilization
- Patient education, ice, treatment of oedema, cautious passive joint mobilization and introduction of gentle active joint exercises
- Patient education and treatment of oedema

**Question 10** – In your clinical practice, which treatment strategies do you mainly prefer during rehabilitation of a patient with TSA?

- Aquatic therapy
- Modalities (e.g., electrotherapy, laser therapy, diathermy)
- Manual therapy and therapeutic exercise with progressive load
- Manual therapy and therapeutic exercise with progressive load, modalities

**Question 11** – In your clinical practice, do you use self-reported outcome measures (self-assessment questionnaires that are filled indirectly by the patient, e.g., DASH) at the beginning and/or end of the rehabilitation treatment after shoulder replacement?

- No
- I don’t know any
- Yes, sometimes
- Yes, always

**Question 12** – In your clinical practice, which non-self-reportedoutcome measures (measures for which the operatorobserves a certain variable and assigns a score, e.g., ROM) do you mainly use to record the obtained results from the treatment of patients after shoulder replacement?

- Strength assessment
- aROM assessment
- pROM assessment
- All previous answers

**SECTION 2 | REHABILITATION AFTER TSA**

**Question 13** – Which movement is important to avoid, as to prevent TSA dislocation?

- Shoulder abduction and external rotation
- Shoulder adduction and internal rotation
- End-range shoulder anterior flexion
- Shoulder internal rotation, adduction and extension

**Question 14** – In your clinical practice, how do you managepROM recovery in patients with TSA?

- pROM from 4th up to 8th week with shoulder forward flexion and external rotation movements
- pROM from 8^th^ up to 12^th^ week with progression, according to patient’s tolerance
- pROM up to first 6weeks, with shoulder forward flexion, abduction, internal rotation and 15°-30° limit of external rotation;full pROM in all direction of movementsfrom 6^th^ to 12^th^ week
- pROM up to first 6 weeks, in all direction of movements; full pROM at 6^th^ week

**Question 15** – In your clinical practice, how do you manage aROM recovery in patients with TSA?

- aROM< 90° of shoulder forward flexion and abduction at 3-4 weeks; aROM> 90° from 6^th^ to 12^th^ weeks; full aROM at 3 months
- assisted aROM with pulley up to first 4 weeks; aROM of shoulder forward flexion from 4^th^ to 8^th^ week; full aROM in all directions of movement with tolerance from 8^th^ to 12^th^ week
- assisted aROM for 6 weeks; full aROM in all directions of movement at 9 weeks
- assisted aROM for 6 weeks; full aROM at 3 months

**Question 16** – In your clinical practice, when do you introduce isometric exercise in patients with TSA?

- 0-3 weeks
- 4-6 weeks; isometric contraction of scapular muscles and distal forearm muscles; isometric contraction in internal and external rotation from 6^th^ to 10^th^ week
- 4-6 weeks
- 5-10 weeks

**Question 17** – In your clinical practice, when do you think it is necessary to start with progressive muscle strengthening in patients with TSA?

- 6-8 weeks
- 9-12 weeks
- Over 12 weeks
- According to patient’s joint recovery

**Question 18** – Which of these muscles are a priority during strengthening phase in patients with TSA?

- Scapular muscles
- Scapular muscles and rotator cuff muscles
- Scapular muscles and deltoid
- Scapular muscles and rotator cuff muscles, deltoid, biceps, triceps

**Question 19** – What is the most common complication that can occur following TSA surgery?

- Infection
- Instability of the glenoid prosthetic component
- Failure of the subscapularis tendon
- Dislocation

**Question 20** – In your clinical practice, following TSA surgery, when should the patient be instructed to mainly return to ADLs (e.g. washing, dressing, combing their hair, cooking)

- 6-9 weeks
- 9-12 weeks
- > 12 weeks
- From week 6 onwards, depending on the patient's recovery (pain reduction, ROM recovery) and the specificity of each activity

**Question 21** – In your clinical practice, following TSA surgery,when do you expect the patient to be able to return to sport? (in details: involving the upper limb, non-contact sportand non-throwing sport)

- 6-12 weeks
- 13-24 weeks
- 7 months -1 year
- Over a year

**SECTION 3 | REHABILITATION AFTER RTSA**

**Question 22** – Which movement is important to avoid, as to prevent RTSA dislocation?

- Shoulder abduction and external rotation
- Shoulder abduction and internal rotation
- End-range shoulder anterior flexion
- Shoulder internal rotation, adduction and extension

**Question 23** – In your clinical practice, how do you manage pROM recovery in patients with RTSA?

- pROM up to first 6 weeks, with 90°-120° shoulder forward flexion and till 30°external rotation with tolerance; full pROM from 6^th^ to 12^th^ week
- pROM from 8^th^ up to 12^th^ week with progression, depending on patient’s tolerance
- pROM up to first 6 weeks; full pROM from 6^th^ to 12^th^ week, included full shoulder external rotation
- No pROM in the first 6 weeks; pROM shoulder movements with tolerance after 6 weeks

**Question 24** – In your clinical practice, how do you manage aROM recovery in patients with RTSA?

- aROM till 90°shoulder forward flexion and 30° external rotation in the first 6 weeks; aROM till 90° of shoulder forward flexion till 12^th^ week; full aROM with tolerance from 12^th^ to 16^th^ week
- aROM till 120° shoulder forward flexion and 30° external rotation in the first 6 weeks; full aROM over 6 weeks
- aROM in all direction of movements with tolerance from 6^th^ week; full aROM from 12^th^ to 16^th^ week
- Hand, wrist and elbow aROM maintenance up to first 6 weeks; full aROM from 12^th^ to 16^th^ week with progression, according to patient’s tolerance

**Question 25** – In your clinical practice, when do you introduce isometric exercise in patients with TSA?

- 0 - 3 weeks
- 4-6 week
- 4 - 6 weeks; isometric contraction of scapular muscles and distal forearm muscles; isometric contraction in internal and external rotation from 6^th^ to 10^th^ week
- >6 weeks

**Question 26** – In your clinical practice, when do you think it is necessary to start with progressive muscle strengthening in patients with RTSA?

- 6-8 weeks
- 9-12 weeks
- Over 12 weeks
- According to patient’s joint recovery

**Question 27** – Which of these muscles are a priority during strengthening phase in patients with RTSA?

- Deltoid
- Rotator cuff muscles
- Scapular muscles
- Deltoid and scapular muscles

**Question 28** – What is the most common complication that can occur following RTSA surgery?

- Scapular notch erosion
- Acromial fracture
- Infection
- Dislocation

**Question 29** – In your clinical practice, following RTSA surgery, when should the patient be instructed to mainly return to ADLs (e.g. washing, dressing, combing their hair, cooking)

- 6-9 weeks
- 9 -12 weeks
- > 12 weeks
- From week 6 onwards, depending on the patient's recovery (pain reduction, ROM recovery) and the specificity of each activity

**Question 30** – In your clinical practice, following RTSA surgery, when do you expect the patient to be able to return to sport? (in details: involving the upper limb, non-contact sport and non-throwing sport)

- 6-12 weeks
- 13-24 weeks
- 7 months -1 year
- Over a year
